# Mental Rotation in American Children: Diminished Returns of Parental Education in Black Families

**DOI:** 10.3390/pediatric12030028

**Published:** 2020-11-20

**Authors:** Shervin Assari

**Affiliations:** 1 Department of Family Medicine, Charles R Drew University of Medicine and Science, Los Angeles, CA 90059, USA; assari@umich.edu; Tel.: +1-734-232-0445; Fax: +1-734-615-8739; 2 Department of Urban Public Health, Charles R Drew University of Medicine and Science, Los Angeles, CA 90059, USA

**Keywords:** age, children, pre-adolescents, mental rotation, cognitive function

## Abstract

***Background:*** While parental education and family socioeconomic status (SES) are associated with an increase in children’s cognitive functioning, and less is known about racial variation in these effects. Minorities’ Diminished Returns (MDRs) suggest that, under racism and social stratification, family SES and particularly parental education show weaker effects on children’s tangible outcomes for marginalized, racialized, and minoritized families, particularly Blacks, compared to Whites. ***Aim:*** We conducted this study to compare the effect of parental education on children’s mental rotation abilities, as an important aspect of cognitive function, by race. ***Methods:*** This cross-sectional study included 11,135 9–10-year-old American children. Data came from baseline of the Adolescent Brain Cognitive Development (ABCD) study. The independent variable was parental education. The dependent variable, mental rotation, was measured by the Little Man Task. Ethnicity, gender, age, marital status, and household income were the covariates. ***Results:*** Parental education was positively associated with mental rotation. However, parental education showed a weaker association with mental rotation in Black than in White families. This was documented by a significant interaction between race and parental education on children’s efficiency score. ***Conclusion:*** Parental education shows a weaker correlation with mental rotation of Black rather than White children, which is probably because of racism, social stratification, and discrimination. This finding is in line with the MDRs phenomenon and suggests that marginalization and racism may interfere with the influences of parental assets and resources and Black American children’s development.

## 1. Introduction

Mental rotation [1], one specific aspect of cognitive performance [2], is the ability to rotate mental representations of two-dimensional and three-dimensional objects within the human mind [3]. Mental rotation is closely associated with abstract thinking, mathematical ability, and spatial memory and analysis [4,5]. Strong mental rotation ability is also associated with better visual perception of complex objects in the space. Mental rotation requires spatial processing, intelligence, abstraction, reasoning, and memory [6]. Mental rotation is commonly being measured by evaluation how well an individual’s brain can easily and efficiently move an objects in three dimensional space [7]. However, studies on social determinants of mental rotation and population variation in mental rotation are rarely done.

The associations between race, socioeconomic status (SES), and cognitive function are among the most sensitive, polarized, and politicalized areas of research in the US [8]. Over the past several decades, there has been an ongoing political debate on whether it is appropriate to study race and cognitive performance, whether race influences cognitive function, and whether such effects are due to social or biological causes [8]. The argument by Murray and others on lower cognitive performance of Black individuals has generated a very strong backlash from the scientific community [9,10]. Questioning racial variation in cognitive performance as a biological and genetic finding, the research community has provided considerable evidence that lower performance of Blacks in cognitive scores reflect poor performance, low education quality, low SES, and other reasons rather than biological difference in this regard [9,10]. Recent research finding that cognitive score predicts the mortality of White but not Black people is another support for the argument that existing cognitive measures fail to capture true cognitive performance of Black children [11]. 

Lack of predictive power of cognitive scores for Black people may also be related to Minorities’ Diminished Returns (MDRs) [12,13]. The MDRs reflect weaker health effects of economic assets of family particularly parental education for any marginalized group such as Black [12,13], Hispanic [14,15,16,17], Asian American [18], Native American [19], lesbian, gay, bisexual, transgender (LGBT) [20], immigrant [21], or even marginalized White [22] people. The non-specific nature of these MDRs suggests that they are not due to biological differences or behaviors, but the way society marginalizes all marginalized groups, including but not limited to Blacks. In this view, if Black children show lower attention, higher impulsivity, or worse educational outcomes, these are not because they are cognitively inferior than Whites but because Black children are sent to worse schools, live in worse neighborhoods, and have experienced high level of stress across all SES levels. That blames the society, rather than Blacks who are themselves the very victim of slavery, racism, segregation, Jim Crow, and unequal treatment [23,24,25]. 

Most of the research on MDRs has focused on the effects of parental education [26], family income [27,28], and marital status [29] on outcomes other than cognitive function. While all these studies have shown that family SES generates fewer developmental, health, emotional, and behavioral outcomes for Black compared to White families [26,27,28,30,31], we are not aware of even a single study on cognitive outcomes other than attention [32] and impulsivity [33,34]. Past work shows that parental SES and particularly parental education shows weaker effects on impulsivity [27], depression [30], anxiety [35], aggression [26], grade point average (GPA) [26,36,37], and substance use [26] for Black rather than White children. As a result of these MDRs, high SES Black children remain at risk of impulsivity [27,34], attention deficit hyperactivity disorder (ADHD) [38], obesity [39], aggression [26], chronic disease [26], anxiety [35], depression [30], and suicide [40], a pattern which does not exist for high SES White children. 

### Aims

Built on MDRs, we compared racial group of children for the effects of parental education on mental rotation, an important aspect of cognitive function. We expected a positive association between parental education and children’s mental rotation; however, we also expected this association to be weaker (diminished) for non-White, particularly Black, rather than White children.

## 2. Methods

### 2.1. Design and Settings

This secondary analysis used a cross-sectional design and borrowed data from the Adolescent Brain Cognitive Development (ABCD) study [41,42,43,44,45]. ABCD baseline data collection was conducted from 2016 to 2018 in 21 sites across states in the United States. For more information on the ABCD study, consult here [41,46].

### 2.2. Participants and Sampling

The ABCD participants were 9–10-year-old children who were selected from multiple cities across the states, US. The ABCD recruitment primarily relied on the US school system. For a detailed description of the sampling and recruitment in the ABCD, consult here [47]. Eligibility for our analysis had valid data on all our study variables including race, age, and mental rotation. The analytical sample of this paper was 11,135.

### 2.3. Study Variables

The study variables included race, ethnicity, sex, age, household income, parental education, marital status, and mental rotation. Race was self-identified: Blacks, Asians, Mixed/Other, and Whites (reference category). Parents reported the age of their children in months. Child sex was a 1 for males and a 0 for females. Parental marital status was reported by the parents and was 1 for married and 0 for other. Household income, reported by the parent, was a three-level categorical measure: less than 50K, 50–100K, and 100+K. 

Mental rotation was evaluated by the Little Man Task (LMT) [48,49,50]. The LMT measures visuospatial processing, flexibility, and attention. This measure is particularly used to measure cognitive aspect that is highly vulnerable to alcohol/drug use. Developed by Acker and Acker (1982) [51], the task involved measurement of visual-spatial processing with varying degrees of difficulty. The LMT is not a memory test. As a part of the task, a rudimentary male figure holding a briefcase in one hand is presented in the middle of the screen. The figure appears in one of the following four positions (1) right side up, (2) upside down, (3) facing the respondent, or (4) with his back to the respondent. The briefcase may be in his right or his left hand. Using the computer buttons, the respondent should indicate which hand holds the briefcase. Left hand, always associated with a button to the left of the participant, is labeled “left”. The result of this test is very sensitivity to visual spatial compromise (e.g., mental rotation). Performance on the LMT is also correlated with the Block Design subtest of the WAIS [51]. In the ABCD, there has been substantial performance variability, with the average percentage of correct trials being 67% (std. dev. = 0.18). The mean reaction time for correct trials was 2670 (+470) ms. 

For the high performers, the average RT for correct trials was 2760 (+368) ms; the numbers for low performers are 2520 (+572) ms. This measure also reflects age-related development. LMT and other cognitive tasks in the ABCD are well explained here [45]. We used the efficiency variable, which is the percentage correct (of the total possible, 32)/average RT to correct responses [45]. This variable is a continuous measure, centered (mean 00.00), and has a higher score reflecting higher mental rotation ability [45].

### 2.4. Data Analysis

**Figure A1:**
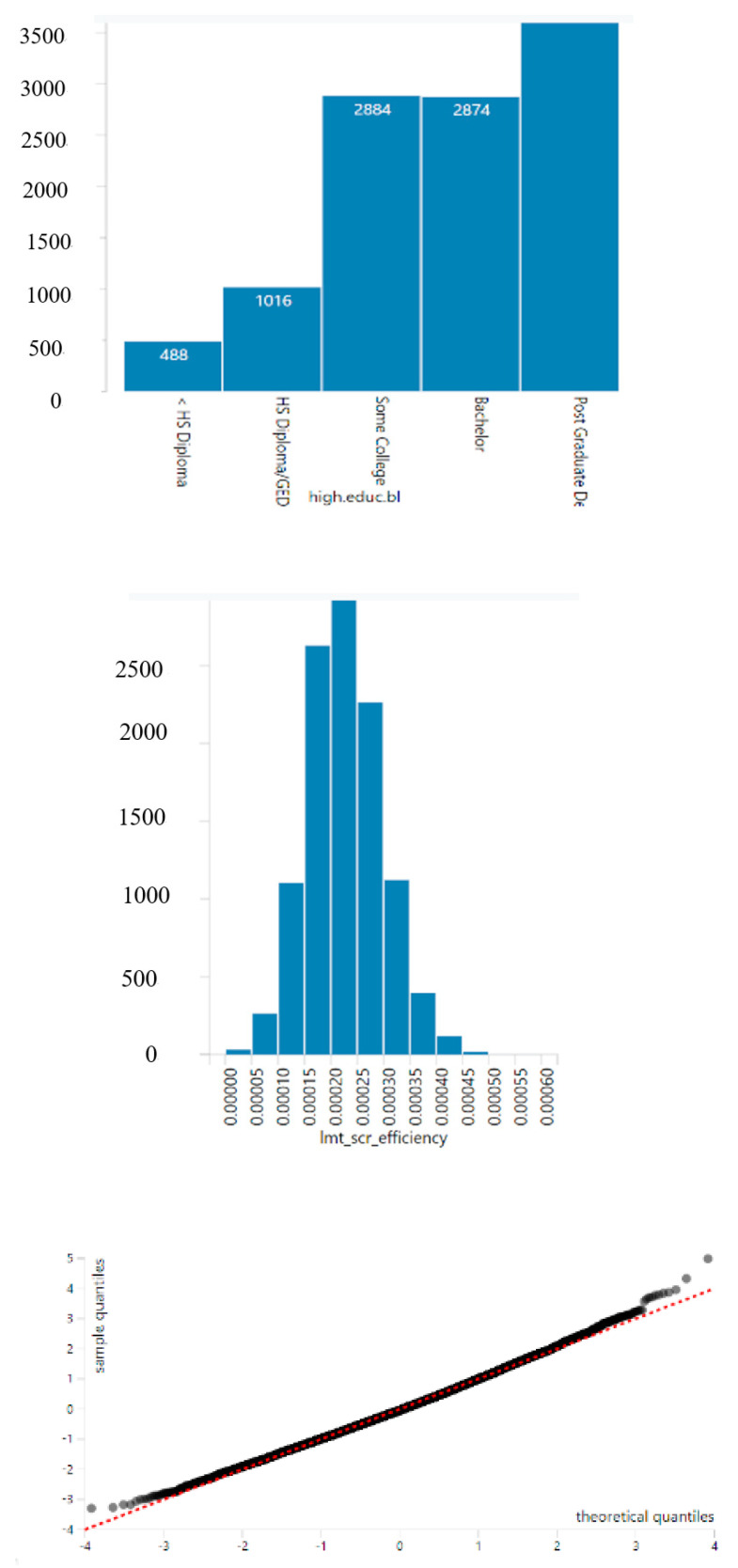

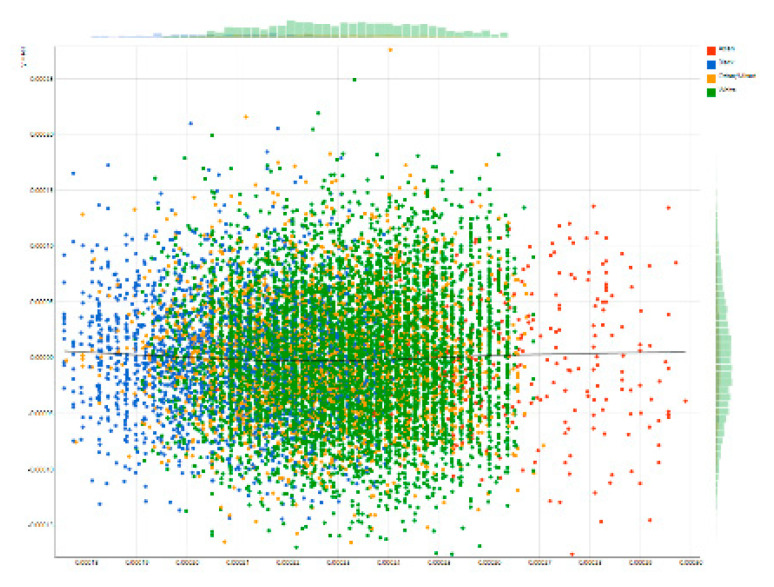
Distribution of predictor, outcome, quantiles, and residuals.

We used Data Exploration and Analysis Portal (DEAP) for data analysis. DEAP provides advanced statistical analysis functions to work with the 2.0 data release from the ABCD study. DEAP is available for the users of the ABCD study. DEAP uses R package for statistical calculations such as linear mixed effects models, while adjusting for the nested nature of the ABCD data. We reported mean (standard deviation (SD)) and frequency (%) of our variables overall and by race. We also performed the Chi-square and Analysis of Variance (ANOVA) for our bivariate analysis. For multivariable modeling, we used mixed-effects regression models that allowed us to adjust for the nested nature of our data. This was because participants are nested to families that are nested to sites and states. Both models were performed in the overall sample. *Model 1* did not have the interaction terms. *Model 2* added interaction terms between race and parental education. In all models, mental rotation (efficiency score), a proxy of cognitive function, was the outcome. Figure A1 shows distribution of our variables and test of regression assumptions. Box A1 shows our models. Regression coefficient (b), SE, *t* value, and *p*-value were reported.

### 2.5. Ethical Aspect

The ABCD study has Institutional Review Board (IRB) approval, and all participants have provided assent or consent, depending their age [46]. Given that our analysis was performed on fully de-identified data, our analysis was exempt from a full IRB review.

## 3. Results

### 3.1. Descriptives

Overall, 11,135, 9–10-year-old children were analyzed. Most participants were Whites (*n* = 7212; 64.8%) followed by Blacks (*n* = 1743 15.7%). From all, 263 were Asian (2.4%) and 1917 (17.2%) were other/mixed race. Table 1 presents the descriptive data overall and by race. This table also compares racial groups for study variables. As this table shows, Black and mixed/other race participants had lowest parental education and White and Asian children had the highest parental education. 

### 3.2. Multivariate Models (Additive Effects)

**Figure 1:**
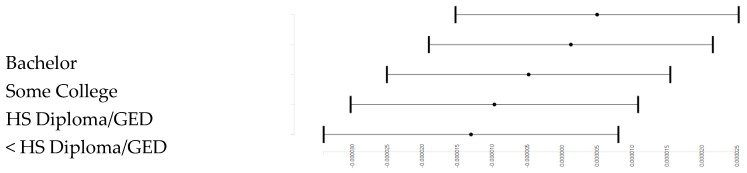
Association between parental education and mental rotation overall.

Table 2 presents the results of two mixed effects regression models in the overall sample. *Model 1* showed a positive association between parental education and mental rotation (Figure 1). 

### 3.3. Multivariate Models (Interactions)

**Figure 2:**
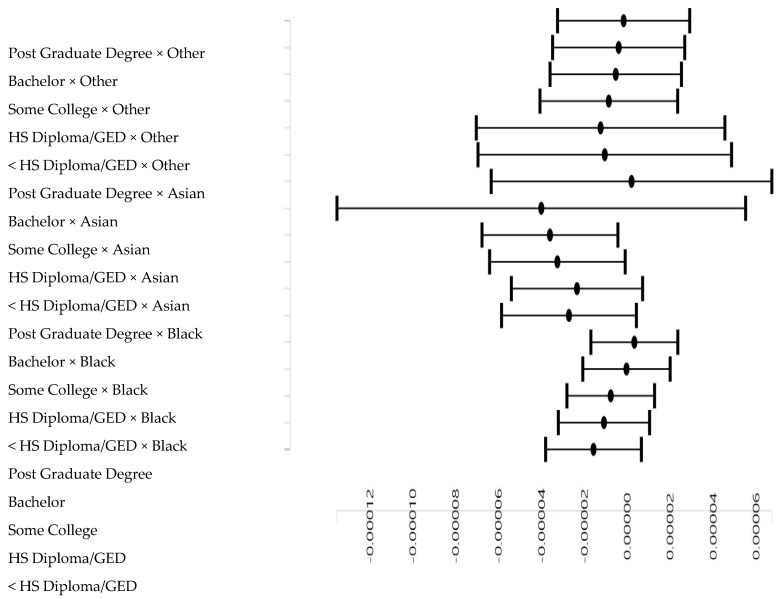
Association between parental education and mental rotation by race.

Table 3 presents the results of a mixed effects regression models in the overall sample. *Model 2* showed an interaction between parental education and race on mental rotation. This interaction indicated that the boosting effect of parental education on mental rotation is weaker for Black than White children (Figure 2). 

## 4. Discussion

This study showed a positive association between parental education and mental rotation overall; however, this was stronger for White than for Black children. That is, while parental education boosts the mental rotation for American children, this effect is weaker in Black than White families. As a result, Black children with highly educated parents remain at low mental rotation, a pattern absent for White children. For White children, children with highly educated parents show highest levels of mental rotation. We did not see similar interactions with other racial groups.

Our finding is in line with MDRs of parental education on mental rotation for Black children. This is fully in line with what is already established on the MDRs of family SES on impulsivity [52], reward responsiveness [53], impulsivity [27], inhibitory control [54], attention [32], and ADHD [38]. Similar MDRs are also reported for the effects of family SES indicators such as parental education, household income, and marital status on behavioral risk such as aggression [26], and substance use [26], as well as mental health risk such as anxiety [35], depression [30], and suicide [40]. These are all diminishing returns of family SES indicators for Black compared to White youth [14,20,55,56]. It is unknown why these MDRs are present for Black children but could not be seen for other racial groups.

These MDRs are not specific to one specific domain or outcome, suggesting that they are due to society but not culture, behavior, or biology. Thus, age does not have a weaker effect on mental rotation of Blacks than Whites because Blacks are innately weaker than Whites. Similarly, the diminished slope is not because Blacks and Whites are biologically different. This is evident because similar MDRs are shown for all marginalized groups with a range of marginalizing identities [12,13]. Thus, they are not specific to Blacks [28] but also Hispanics [14,15,16,17], Asian Americans [18], Native Americans [19], LGBTQs [20], immigrants [21], or even marginalized Whites [22]. They are also not specific to a particular age group, as documented for children [27,28,31], adults [55], and older adults [57]. Finally, these MDRs are relevant to economic resources such as SES [14,27,35,58,59], and non-economic assets such as self-efficacy [60,61]. This paper extends the previous work on MDRs to the area of cognitive function.

A wide range of sociological and economic mechanisms explain the MDRs of human capital and economic resources on mental rotation for Black related to White families. Black families experience high levels of stress across all SES levels [62]. Social mobility is more taxing for Black than White families [63]. At all SES levels, exposure [64,65,66,67,68] and vulnerability [58] to discrimination is high for Black families. While low SES Black families struggle with food insecurity and poverty and neighborhood disorder, high SES Black families experience discrimination due to proximity to Whites [64,65]. As discrimination reduces the chance of healthy brain development [58,67,69], Black children may remain at risk of impulsivity across the whole SES spectrum. As a result, age, the main driver of development, shows weaker effects for Black than White children.

While low SES and poor outcomes are two types of disadvantage in Black communities, MDRs reflect a qualitatively different set of disadvantage [12,13]. While the former is reflective of unequal outcomes and opportunities, the latter is reflective of low response to the presence of individual level resources. It is due to the latter that policymakers may observe sustained inequality despite investments. To address the latter, there is a need to address systemic causes of inequalities. As a result of these two jeopardies, Blacks are experiencing a double disadvantage, where not only resources are scarce, the influence of the individual level resources and assets are dampened, given the environment [12,70].

Multilevel economic and environmental mechanisms are in play that reduce the marginal returns of family SES. MDRs are attributed to social stratification, racism, and marginalization. These processes function across multiple societal institutions [12,70]. Racial injustice, prejudice, and discrimination have historically interfered with the gain of resources and assets for the Black communities [71,72,73]. One of many causes of MDRs may be childhood poverty [74]. As a result of such environmental and structural injustice, we observe MDRs across resources, assets, outcomes, settings, and age groups.

### Limitations

The current study has some methodological shortcomings. First, because of a cross-sectional design, it is inappropriate for us to draw any causal inferences. However, age is a known determinant of brain development. So, the direction of the association between age and mental rotation is from age to cognitive performance not vice versa. Still, the findings reported here are correlations, not causes. To established stronger causal evidence, we need to use longitudinal data and map changes in cognitive function with increase in age over time. Our expectation is that process of aging is better associated with cognitive enhancement for White than Black children. Similarly, we only tested the MDRs of parental education. Previous work had established MDRs of family SES on non-cognitive outcomes [27,33,52,53]. In addition, we only focused on family SES. It is imperative to control for contextual and neighborhood level indicators as well as health. Finally, we did not study how these MDRs change over time. 

## 5. Conclusions

Relative to their White counterparts, Black children show weaker effects of parental education on mental rotation. This is important because mental rotation and cognitive function are drivers for a wide range of educational outcomes. To minimize the Black–White gap in brain development, there is a need to address societal barriers that cause MDRs of economic and non-economic resources in Black communities and families. We need economic, public, and social policies that go beyond individual-level risk factors and address systemic, structural, and societal causes of inequalities.

## Figures and Tables

**Table 1:** Descriptive characteristics overall and by race (*n* = 11,135).

	Level	All	White	Black	Asian	Other/Mixed	*p*
*n*		11,135	7212	1743	263	1917	
Parental Education	<HS Diploma	488 (4.4)	200 (2.8)	147 (8.4)	7 (2.7)	134 (7.0)	<0.001
	HS Diploma/GED	1016 (9.1)	369 (5.1)	422 (24.2)	3 (1.1)	222 (11.6)	
	Some College	2884 (25.9)	1524 (21.1)	689 (39.5)	17 (6.5)	654 (34.1)	
	Bachelor	2874 (25.8)	2114 (29.3)	250 (14.3)	74 (28.1)	436 (22.7)	
	Post Graduate Degree	3873 (34.8)	3005 (41.7)	235 (13.5)	162 (61.6)	471 (24.6)	
Sex	Female	5339 (47.9)	3389 (47.0)	885 (50.8)	137 (52.1)	928 (48.4)	0.017
	Male	5796 (52.1)	3823 (53.0)	858 (49.2)	126 (47.9)	989 (51.6)	
Married Family	No	3529 (31.7)	1527 (21.2)	1224 (70.2)	36 (13.7)	742 (38.7)	<0.001
	Yes	7606 (68.3)	5685 (78.8)	519 (29.8)	227 (86.3)	1175 (61.3)	
Hispanic	No	8941 (80.3)	5921 (82.1)	1663 (95.4)	243 (92.4)	1114 (58.1)	<0.001
	Yes	2194 (19.7)	1291 (17.9)	80 (4.6)	20 (7.6)	803 (41.9)	
		Mean (SD)	Mean (SD)	Mean (SD)	Mean (SD)	Mean (SD)	
Age		118.97 (7.47)	119.04 (7.50)	118.88 (7.29)	119.46 (7.75)	118.69 (7.47)	0.196
Mental Rotation		0.00 (0.00)	0.00 (0.00)	0.00 (0.00)	0.00 (0.00)	0.00 (0.00)	<0.001

**Table 2:** Mixed effects regressions overall (Model 1).

Characteristics	*b*	*SE*	*t*	*p*
Parental Education (HS Diploma/GED)	0e + 00	0.00	0.88	0.380
Parental Education (Some College)	1e-05 *	0.00	2.41	0.016
Parental Education (Bachelor)	1e-05 ***	0.00	4.02	<0.001
Parental Education (Post Graduate Degree)	2e-05 ***	0.00	5.09	<0.001
Race (Black)	−2e-05 ***	0.00	−7.38	<0.001
Race (Asian)	3e-05 ***	0.00	7.70	<0.001
Race (Other/Mixed)	0e + 00	0.00	-0.84	0.402

* *p* < 0.05. ** *p* < 0.01. *** *p* < 0.001.

**Table 3:** Mixed effects regressions overall (Model 2).

Characteristics	*b*	*SE*	*t*	*p*
Parental Education (HS Diploma/GED)	0e + 00	0.00	0.81	0.419
Parental Education (Some College)	1e-05	0.00	1.54	0.125
Parental Education (Bachelor)	2e-05 **	0.00	2.93	0.003
Parental Education (Post Graduate Degree)	2e-05 ***	0.00	3.66	0.000
Race (Black)	0e + 00	0.00	−0.54	0.589
Race (Asian)	3e-05	0.00	1.11	0.268
Race (Other/Mixed)	−1e-05	0.00	−1.59	0.113
Parental Education (HS Diploma/GED) × Race (Black)	−1e-05	0.00	−1.28	0.200
Parental Education (Some College) × Race (Black)	−1e-05	0.00	−0.95	0.344
Parental Education (Bachelor) × Race (Black)	−2e-05	0.00	−1.90	0.057
Parental Education (Post Graduate Degree) × Race (Black)	−2e-05 *	0.00	−2.28	0.022
Parental Education (HS Diploma/GED) × Race (Asian)	−2e-05	0.00	−0.52	0.603
Parental Education (Some College) × Race (Asian)	2e-05	0.00	0.58	0.562
Parental Education (Bachelor) × Race (Asian)	1e-05	0.00	0.19	0.846
Parental Education (Post Graduate Degree) × Race (Asian)	0e+00	0.00	0.13	0.900
Parental Education (HS Diploma/GED) × Race (Other/Mixed)	1e-05	0.00	0.75	0.455
Parental Education (Some College) × Race (Other/Mixed)	1e-05	0.00	1.26	0.208
Parental Education (Bachelor) × Race (Other/Mixed)	1e-05	0.00	1.39	0.163
Parental Education (Post Graduate Degree) × Race (Other/Mixed)	1e-05	0.00	1.69	0.092
Race (Other/Mixed)	0e + 00	0.00	−0.84	0.402

* *p* < 0.05. ** *p* < 0.01. *** *p* < 0.001.

## Appendix A

Box A1 Model Formula.
**Model 1**
lmt_scr_efficiency ~ high.educ.bl + race.4level + sex + married.bl + age + hisp Random: ~(1|rel_family_id)
**Model 2**
lmt_scr_efficiency ~ high.educ.bl + race.4level + sex + married.bl + age + hisp + race.4level high.educ.bl Random: ~(1|rel_family_id)
